# Frequency of microsatellite instability in gynecologic cancers and the efficacy of immune checkpoint inhibitors treated: real-world data from a single gynecologic center

**DOI:** 10.3389/fimmu.2025.1567824

**Published:** 2025-05-09

**Authors:** Wei Kuang, Jing Zeng, Lingling Tong, Qianqi Liu, Huanxin Sun, Min Feng, Dongni Liang, Wei Wang, Cheng Wang

**Affiliations:** ^1^ Department of Pathology, West China Second University Hospital of Sichuan University, Chengdu, Sichuan, China; ^2^ Key Laboratory of Birth Defects and Related Diseases of Women and Children, Sichuan University, Chengdu, Sichuan, China; ^3^ Department of Obstetrics and Gynecology, West China Second University Hospital of Sichuan University, Chengdu, Sichuan, China

**Keywords:** microsatellite instability, gynecologic cancers, immunotherapy, immune checkpoint inhibitors (ICIs), objective response rate (ORR)

## Abstract

**Objective:**

This study evaluated the incidence of Microsatellite Instability-High (MSI-H) in patients with gynecologic cancers in a single gynecologic center and investigated the effect of immune checkpoint inhibitors (ICIs) in treating MSI-H in advanced or recurrent gynecologic cancers.

**Methods:**

We conducted a retrospective study of patients diagnosed with gynecological cancers between June 2021 and May 2024. We investigated their clinicopathological information, the results of microsatellite instability (MSI), the immunohistochemistry staining PD-L1 analyses, the molecular classification testing, and the tumor response to treatment with ICIs.

**Results:**

Among 1333 patients included in the analysis, the frequency of MSI-H was 1.3% (3/223) in cervical cancer, 25.7% (280/1091) in endometrial cancer, and 10.5% (2/19) in ovarian or tubal and peritoneal cancer. When the patients were evaluated by histologic type, the frequency of MSI-H was 26.1% (241/921) in endometrioid adenocarcinoma and 35.1% (20/57) in mixed adenocarcinoma. Molecular classification results for the 1020 cases that successfully underwent the tests were 71 for the POLE mutation (POLEmut) subtype, 271 for MMR-deficiency (MMRd) subtype, 571 for the non-specific molecular profile (NSMP) subtype, and 107 for the p53 abnormality (p53abn) subtype. Thirty-five patients were treated with ICIs for at least one cycle. The objective response rate (ORR) was 34.3% (95% CI, 19.1% to 52.2%). Among the patients who achieved an objective response, the median time to respond was 2.65 months, and the median duration of response had not been reached. The median progression-free survival (PFS) was 9 months (95% CI, 4 to 10), and the median overall survival (OS) had not been reached. Additionally, in the patients with endometrial cancer, the median PFS in MSI-H patients was 5 months versus 3 months in microsatellite stable (MSS) patients (Δ = 2 months; p=0.92), and the median OS in both MSI-H and MSS patients had not been reached (p=0.89).

**Conclusion:**

This study had shown the MSI-H frequencies for the three major types of gynecological tumors and demonstrated the clinical benefit of treatment with ICIs in patients with advanced or recurrent gynecologic cancer. Among endometrial cancer patients, the effects of immunotherapy may be consistent regardless of MSI status.

## Introduction

In recent years, immune checkpoint inhibitors (ICIs) have undoubtedly been one of the successful tumor immunotherapies, and their rapid development has revolutionized oncology treatment, leading to a change in solid tumor therapy (1, 2). However, immunotherapy offers new treatment options for patients with advanced/recurrent gynecological tumors compared to conventional therapies. Programmed cell death protein 1 (PD-1) and its ligand (PD-L1) inhibitors are ineffective or have a short efficacy duration in most patients due to primary or secondary resistance, a bottleneck for immunotherapy. Therefore, finding more effective molecular predictive markers would benefit the future development of immunotherapy. Microsatellite instability (MSI) status is now widely used as a screening tool to identify Lynch syndrome, which is associated with an increased risk of both endometrial and ovarian cancers (3) and as one of the predictive markers of efficacy in ICIs (4–6).

Normal cells can detect and repair base mismatches that arise during DNA replication and recombination, usually by the four common mismatch repair (MMR) proteins, MLH1, MSH2, MSH6, and PMS2 (7). When MMR genes are mutated, or the promoter of the MLH1 gene is methylated, it is possible to result in deficiency-MMR (dMMR) (8). Then, the dMMR can accumulate DNA replication errors, leading to microsatellite instability-high (MSI-H) (9). MSI-H/dMMR tumors share similar histopathological features, including somatic hypermutation, increased neoantigen formation, more lymphocytic infiltration, and strong expression of immune checkpoint proteins (e.g., PD-L1, PD-1). They correlate strongly with a high or low tumor mutational burden (TMB) degree. Neoantigens generated through MSI-H/dMMR may become targets for immunotherapy, activating the immune system through immune checkpoint inhibitors, which then modulate their anti-tumor effects (10, 11).

MSI-H/dMMR is associated with multiple types of gynecologic malignancies. Among patients with gynecologic cancers, the frequency of endometrial cancer ranges from 20% to 40% (12), ovarian cancer from 1% to 3%, and cervical cancer from 2% to 4% (13–15). The Food and Drug Administration (FDA) granted accelerated approval for Pembrolizumab in 2017 for treating a wide range of unresectable or metastatic MSI-H/dMMR solid tumors (5). Some follow-up studies have shown a high treatment response rate (16–18). Recently, the National Medical Products Administration (NMPA) has successively approved several domestic PD-1/PD-L1 inhibitors, such as tislelizumab, for the pan-oncological MSI-H/dMMR indication. RATIONALE-209 is a single-arm, open-label, multi-center Phase II study to evaluate the efficacy and safety of tislelizumab monotherapy in patients with treated, locally advanced, unresectable, or metastatic MSI-H/dMMR solid tumors and is the first clinical study to publish subgroup data from a Chinese population-based MSI-H/dMMR gynecological tumor subgroup. The results showed that the objective response rate (ORR) of tislelizumab in MSI-H/dMMR gynecological tumors was 53.3%, including 46.2% in patients with endometrial cancer, and the efficacy of tislelizumab was assessed to be PR in patients with ovarian and cervical cancers (19). Particularly in endometrial cancer, about 20%-30% of patients have dMMR/MSI-H status, and these patients respond better to immunotherapy. For patients with proficient mismatch repair (pMMR)/microsatellite instability-stable (MSS), the efficacy of immunotherapy is unclear or controversial. A meta-analysis study showed that only combining anti-PD-1 agents with chemotherapy resulted in a PFS benefit in pMMR patients (20). Another meta-analysis showed that, when stratified by MMR status, patients with pMMR only had an improvement in PFS (HR=0.74) but did not reach statistical significance in OS (HR=0.86) (21).

Although immunotherapy is currently effective in randomized trials, results from retrospective immunotherapy studies in gynecological tumors are still limited. In this study, we retrospectively evaluated the incidence of MSI-H in patients with gynecologic cancers in our center and investigated the effect of ICIs in treating advanced or recurrent gynecologic cancers with MSI-H status. We also focused on the difference in efficacy for patients with advanced or recurrent endometrial cancer with MSS or MSI-H status.

## Materials and methods

### Study design and patients

**Figure 1:**
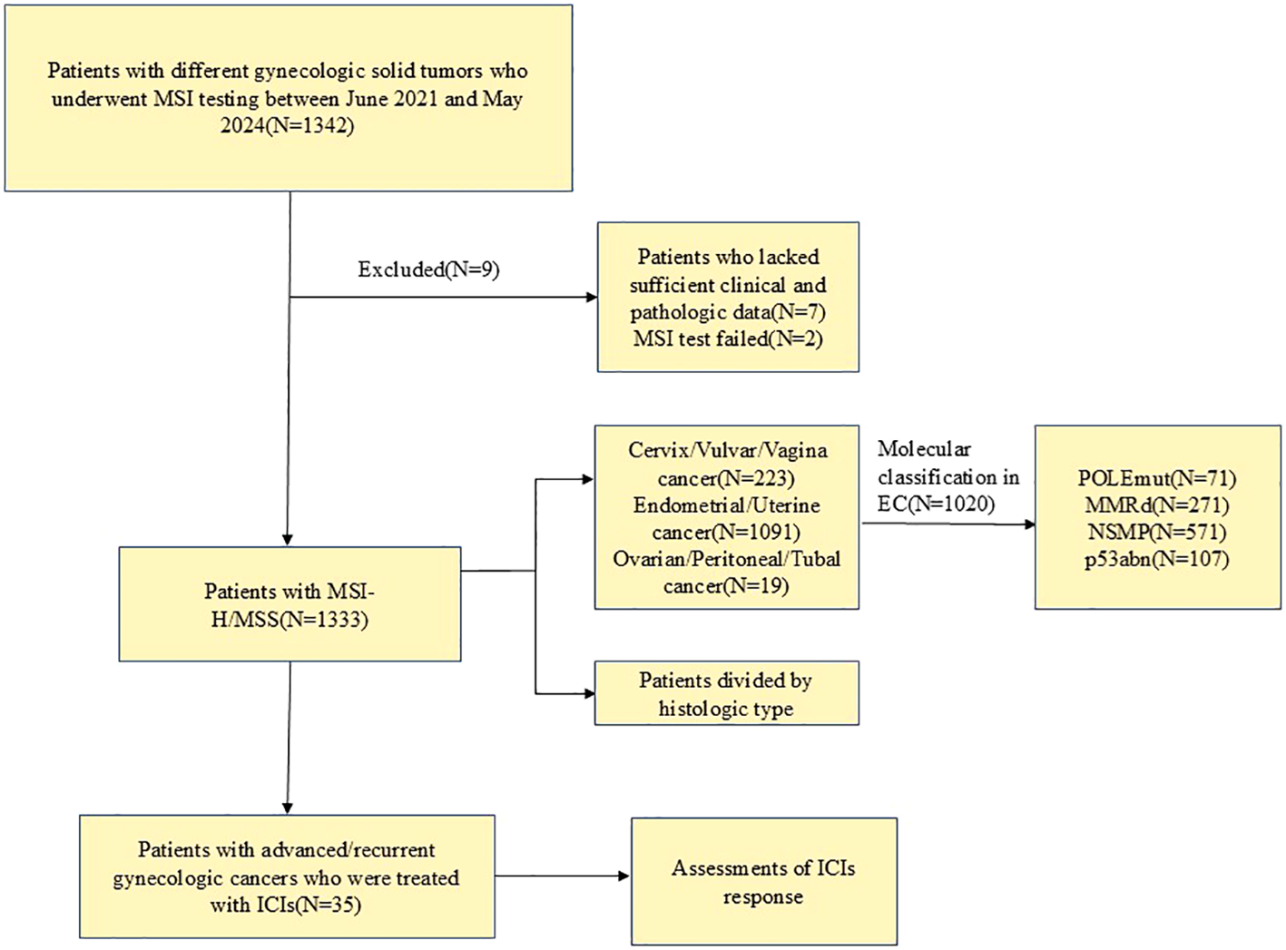
Flow diagram of patient selection. MSI, microsatellite instability; MSI-H, microsatellite instability-high; MSS, microsatellite stable; ICIs, immune checkpoint inhibitors; EC, Endometrial cancer; POLEmut, POLE mutation; MMRd, MMR deficiency; NSMP, non-specific molecular profile; p53abn, p53 abnormality.

We retrospectively summarized the medical records of patients with different gynecologic solid cancers who underwent MSI testing at the Second West China Hospital of Sichuan University between June 2021 and May 2024. A total of 1342 patients were tested for MSI, of which seven were excluded because no clinical information was available for questioning after the external consultation, 2 MSI tests failed due to DNA quality problems, and 1333 cases were successfully enrolled. We further reviewed the clinicopathological and radiological records of patients who were diagnosed with advanced/recurrent gynecological cancers and treated with at least one cycle of ICIs. The patient inclusion criteria are shown in a flow diagram in **Figure 1**. Patients receive tislelizumab/pembrolizumab 200 mg intravenously (IV) every 3 weeks until the condition worsens, an intolerable toxic reaction occurs, the physician makes a decision, or the patient withdraws consent. The study was approved by the Ethics Committee of West China Second University Hospital of Sichuan University Institutional [No.2024 (181)], and conducted according to Helsinki’s Declaration.

### Microsatellite instability testing

The UPure formalin-fixed paraffin-embedded (FFPE) Tissue DNA Kit (Biokeyston, Chengdu, China) was used to extract DNA from tissues with enriched tumor and non-tumor areas. Based on the recommendations in the Bethesda guidelines, the MSI testing kit (Tongshu, BioTech, Changzhou, China) with five markers (BAT25, BAT26, D2S123, D5S346, D17S250) was used to compare the microsatellite status of tumor and normal tissue DNA. MSI-H is defined as instability in ≥ 2 loci, low microsatellite instability (MSI-L) is defined as instability in one locus, and MSS is defined as no instability in the five loci. Two experienced molecular pathologists carried out the interpretation.

### Immunohistochemical analysis

The slides with four µm-thick tissue sections were stained with PD-L1 immunohistochemical 22C3 antibody (Agilent Technologies, Inc., Santa Clara, CA) and p53(clone MX056, Fuzhou Maxin, China) at our institution. The measure of PD-L1 expression was the combined positive score (CPS), defined as the ratio of PD-L1–positive cells (tumor cells, lymphocytes, and macrophages) to the total number of tumor cells multiplied by 100. PD-L1 positivity was defined as a CPS of 1 or greater. Criteria for interpreting p53 immunostaining were used as described previously (22).

### Molecular classification testing

At our center, we used immunohistochemistry (IHC) for p53, MSI assay, and Sanger sequencing to detect pathogenic variants in the structural domain of the POLE exonuclease (primer sequences are available upon request) based on the Proactive molecular risk classifier for endometrial cancer (ProMisE) assay strategy. We classified the endometrial cancers into four distinct molecular subgroups: POLE mutation (POLEmut), MMR deficiency (MMRd), p53 abnormality (p53abn), or no specific molecular profile (NSMP) (23, 24). MMR IHC and molecular testing for MSI are used to detect MMR defects. These two approaches have comparable sensitivity and are shown to be ~95% concordant (25). Therefore, we consider the results of the MSI assay and the MMR IHC equivalent if we treat the MSS/MSS-L as a pMMR and the MSI-H as a dMMR.

### Outcomes

Baseline tumor assessment before treatment initiation and response is assessed by abdominopelvic and/or chest computed tomography scans at least every 9 weeks. Response evaluation criteria in solid tumor (RECIST1.1) and immunotherapy-related RECIST (irRECIST) were utilized to evaluate tumor response (26, 27). The primary endpoint was ORR, defined as the proportion of patients with complete response (CR) or partial response (PR). Secondary endpoints include time to response (TTR) and duration of response (DOR), the time from the first occurrence of CR or PR to disease progression or death, whichever occurs first. Progressive-free survival (PFS) is defined as the time from the first dose of ICI treatment to tumor progression or death, whichever occurs first. Overall survival (OS) is defined as the time from the first dose of ICI treatment to death from any cause. Efficacy and safety analyses included all patients who received at least one cycle of ICIs.

### Statistical analysis

Demographic and baseline characteristics and response measures were summarized using descriptive statistics or league tables. ORR point estimates were accompanied by 95% CIs using the Clopper-Pearson exact method based on the binomial distribution. Patients without response data were considered non-responders. The Kaplan-Meier method estimated the duration of response, PFS, and OS. All statistical analyses were performed using R software (version 4.2.1). A p-value of <0.05 was considered statistically significant.

## Results

### Patients and the frequency of MSI-H in gynecologic cancers

A total of 1335 patients with gynecologic cancers were included in the analysis. Due to insufficient DNA quality, 2 of the patient samples failed the MSI test. One patient underwent MSI testing twice because of two synchronous primary cancers. Patients were categorized into MSI-H and MSI-L/MSS based on MSI status to compare baseline clinicopathologic parameters. The specific results are shown in **Supplementary Table S1**. Most patients (71.2%, 949/1333) were aged over 50 years; Endometrial/uterine cancer accounted for the highest proportion of cases included in this study, at 81.8% (1091/1333). This cancer type was also the highest in the MSI-H population, with a high percentage of 98.2% (280/285). The majority of patients (72.7%, 969/1333) were diagnosed with a tumor International Federation of Gynecology and Obstetrics (FIGO) stage I-II, and 74.9% (998/1333) had a tumor of G1/2.

**Figure 2:**
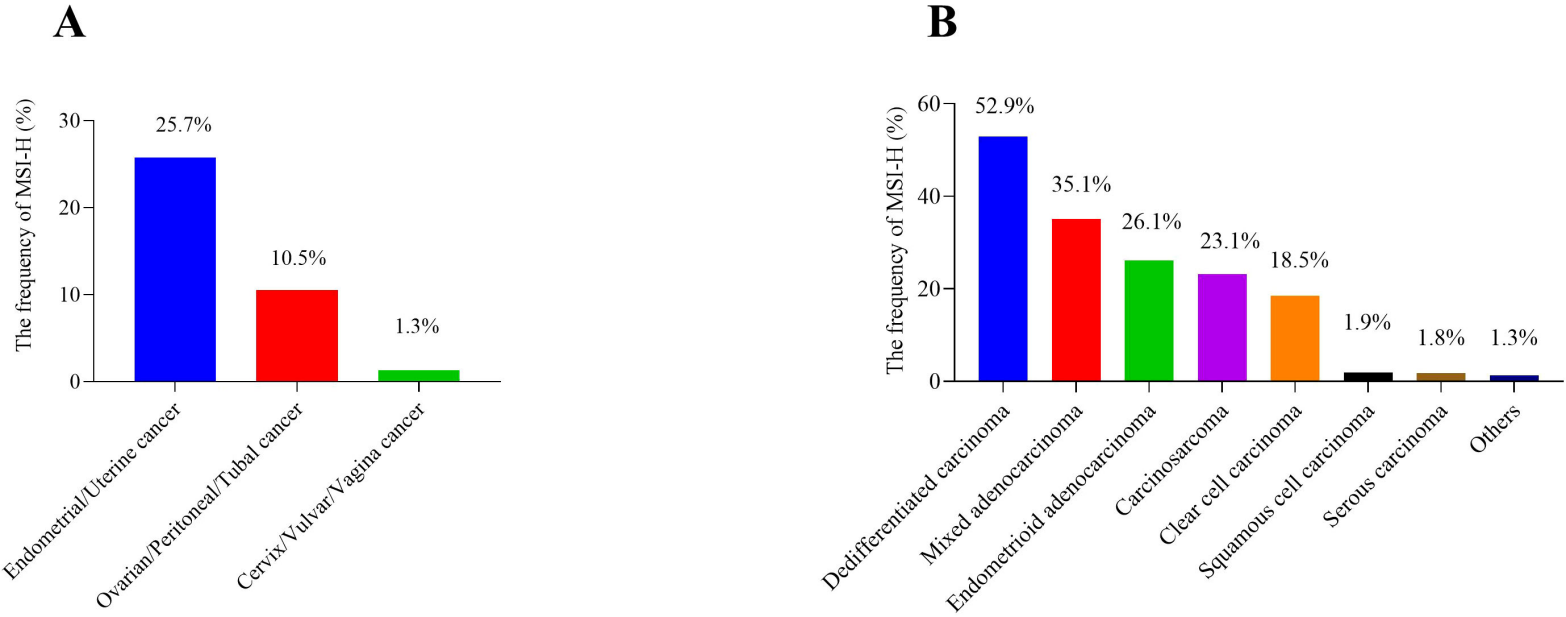
The frequency of MSI-H in gynecologic cancers. **(A)** divided by different origins of cancers; **(B)** divided by different histologic types. MSI-H, microsatellite instability-high.

By the origin of cancer, the frequency of MSI-H was 1.3% (3/223) in cervix/vulvar/vagina cancer, 25.7% (280/1091) in endometrial/uterine cancer, 10.5% in ovarian/peritoneal/tubal cancer (2/19) (**Figure 2A**). Regardless of the origin of cancer not considered, when the patients were evaluated by histologic type, the frequency of MSI-H was 23.1% (6/26) in carcinosarcoma, 26.1% (240/920) in endometrioid adenocarcinoma, 35.1% (20/57) in mixed adenocarcinoma, 1.8% (1/54) in serous carcinoma, 1.9% (3/157) in squamous cell carcinoma, 52.9% (9/17) in dedifferentiated carcinoma which is the highest, and 18.5% (5/27) in clear cell carcinoma, but in other types such as adeno-squamous carcinoma, neuroendocrine carcinoma, etc. the frequency was only 1.3% (1/77) (**Figure 2B**).

Out of 1091 endometrial cancers, 1024 cases underwent molecular classification tests, of which 4 cases could not be typed due to failure of the POLE sequencing test. Molecular classification results were 71 for POLEmut, 271 for MMRd, 571 for NSMP, and 107 for P53abn among the 1020 successfully molecularly classified cases (**Supplementary Table S2**).

### Baseline clinicopathologic characteristics of patients treated with ICIs

As shown in **Table 1**, 35 patients were treated with at least one ICI, and 16 were treated after recurrence. Among them, the median age of patients was 54 [Inter Quartile Range (IQR), 48 to 58], and 82.9% (29/35) had FIGO stage III or FIGO stage IV disease at the initial diagnosis. In total, the most common type represented among the patients is cervical cancer (n=22), followed by endometrial cancer (n=11), and the remaining two cases were vulvar cancer. There were 31% (11/35) cases of MSI-H tumors. Others were in MSS status. Only 6 out of 11 endometrial cancers were analyzed for molecular classification. Only 1 case was the p53abn subtype, and the other five were the MMRd subtype. PD-L1 expression was assessed in 28 patients, 27 (96.4%) of whom were PD-L1 positive. The patients with MSS status were also treated with ICIs because their tumors expressed PD-L1 or their histology matched that of patients who responded to ICIs. The specific drugs of the ICIs used were pembrolizumab (22.9%, 8/35) and tislelizumab (77.1%, 27/35). As of 25 December 2024, the median follow-up was 17 months (1 to 56 months). Seventeen patients (48.6%) discontinued ICIs, most commonly due to disease progression or death. Patients received a median of 8 cycles of chemotherapy with ICIs (ranging from 1 to 29 cycles).

**Table 1:** Baseline characteristics of patients treated with ICIs.

Characteristic	n (%)
Age	
Median (IQR)	54 (48-58)
Range	33-74
FIGO stage	
I-II	6 (17.1)
III-IV	29 (82.9)
Origin of cancer	
CC	22 (62.9)
EC	11 (31.4)
VC	2 (5.7)
Histology	
Endocervical adenocarcinoma	2 (5.7)
Carcinosarcoma	1 (2.9)
Clear cell carcinoma	1 (2.9)
Dedifferentiated carcinoma	2 (5.7)
Endometrioid adenocarcinoma	7 (20)
serous carcinoma	1 (2.8)
Squamous cell carcinoma	21 (60)
PD-L1 expression status	
Positive	27 (77.1)
Negative	1 (2.9)
Unknown	7 (20)
MSI status	
MSI-H	11 (31.4)
MSS	24 (68.6)
Molecular classification*	
POLEmut	0 (0)
MMRd	5 (45.5)
NSMP	0 (0)
p53abn	1 (0.09)
NA	5 (45.6)
Previous radiotherapy	
yes	32 (91.4)
no	2 (5.7)
Unknown	1 (2.9)
Previous antineoplastic agents	
Paclitaxel	35 (100)
Cisplatin	15 (42.9)
Carboplatin	23 (65.7)
Bevacizumab	12 (34.2)
Oxaliplatin	4 (11.4)
Gemcitabine	1 (2.8)
Lenvatinib	1 (2.8)
Nedaplatin	1 (2.8)
No. of previous lines of chemotherapy	
1	17 (48.5)
2	16 (45.7)
≥3	2 (5.7)
Type of immune checkpoint inhibitor	
Pembrolizumab	8 (22.9)
Tislelizumab	27 (77.1)

ICIs, immune checkpoint inhibitors; IQR, Inter Quartile Range; FIGO, International Federation of Gynecology and Obstetrics; CC, Cervical cancer; EC, Endometrial cancer; VC, Vulval cancer; PD-L1, programmed death-ligand 1; MSI-H, microsatellite high; MSS, microsatellite stable; POLEmut, POLE mutation; MMRd, MMR deficiency; NSMP, non-specific molecular profile; p53abn, p53 abnormality; NA, no available.

*Molecular classification testing is only available for Endometrial Cancer.

### Antitumor activity

In the total population (n=35), five patients (14.3%) had a confirmed complete response, and 7 (20.0%) had a confirmed partial response, resulting in an ORR of 34.3% (95% CI, 19.1% to 52.2%) (**Table 2**). According to the origin of the tumor, the ORR was 40.9% for cervical cancer, 18.2% for endometrial cancer, and 50% for vulvar cancer (**Table 3**). Among patients with MSI-H tumors (n=11), the ORR was 36.4% (3 CRs and 1 PR). The ORRs were 25% (2/8) for endometrial cancer with MSI-H and 66.7% (2/3) for cervical cancer with MSI-H (**Table 4**). The ORR for patients treated with Pembrolizumab was 37.5% (3/8) compared to 33.3% (9/27) for patients treated with Tislelizumab (**Table 5**). Among the patients who achieved an objective response, the median time to response was 2.65 months (range, 0.8 to 7.0 months), the median duration of response had not been reached (range, 7 to 30+ months), and the 12-month DOR rate was 65.6% (95%CI, 32% to 85.5%) (**Table 2**).

**Table 2:** Tumor responses assessed by RECIST v.1.1 and irRECIST.

Antitumor activity	Total (n=35)	MSI-H (n=11)	MSS (n=24)
Best overall response			
CR	5 (14.3)	3 (27.3)	2 (8.3)
PR	7 (20)	1 (9.1)	6 (25.0)
SD	7 (20)	2 (18.2)	5 (20.8)
PD	10 (28.6)	3 (27.3)	7 (29.2)
Not able to be assessed*	6 (17.1)	2 (18.2)	4 (16.7)
ORR	12 (34.3)	4 (36.4)	8 (33.3)
95%CI	19.1-52.2	10.9-69.2	15.6-55.3
DCR	19 (54.3)	6 (54.5)	13 (54.2)
95%CI	36.6-71.2	23.4-83.3	32.8-74.4
TTR (mo)			
Median(95%CI)	2.65 (1.7-4.0)	2.85 (2.4-4)	2.2 (0.8-7.0)
Range	0.8-7	2.4-4	0.8-7
DOR (mo)			
Median	NR	NR	NR
Range	7 to ≥30	9 to ≥30	7 to 14

Data are presented as No. (%) unless otherwise noted. RECIST, response evaluation criteria in solid tumors; irRECIST, immunotherapy related RECIST; CR, complete response; PR, partial response; SD, stable disease; PD, progressive disease; ORR, objective response rate; DCR, disease control rate; TTR, time to response; DOR, duration of response; NR, not reached.

*Patients who had no postbaseline tumor assessment because of death, withdrawal of consent, loss to follow-up, or start of new anticancer therapy.

**Table 3:** Antitumor activity of ICIs with different origins of cancer.

Origins of cancer	No.	CR, n (%)	PR, n (%)	ORR (%)
Cervical cancer	22	3 (13.6)	6 (27.3)	40.9
Endometrial cancer	11	2 (18.2)	0	18.2
Vulvar cancer	2	0	1 (50)	50

ICIs, immune checkpoint inhibitors; CR, complete response; PR, partial response; ORR, objective response rate.

**Table 4:** Antitumor activity of ICIs in MSI-H cancers.

Origins of cancer	No.	CR, n (%)	PR, n (%)	ORR (%)
Cervical cancer	3	1 (33.3)	1 (33.3)	66.6
Endometrial cancer	8	2 (25)	0	25.2

ICIs, immune checkpoint inhibitors; MSI-H, microsatellite high; CR, complete response; PR, partial response; ORR, objective response rate.

**Table 5:** Antitumor activity of different types of ICIs.

Type of ICIs	No.	CR, n (%)	PR, n (%)	ORR (%)
Pembrolizumab	8	2 (25.0)	1 (12.5)	37.5
Tislelizumab	27	3 (11.1)	6 (22.2)	33.3

ICIs, immune checkpoint inhibitors; CR, complete response; PR, partial response; ORR, objective response rate.

**Figure 3:**
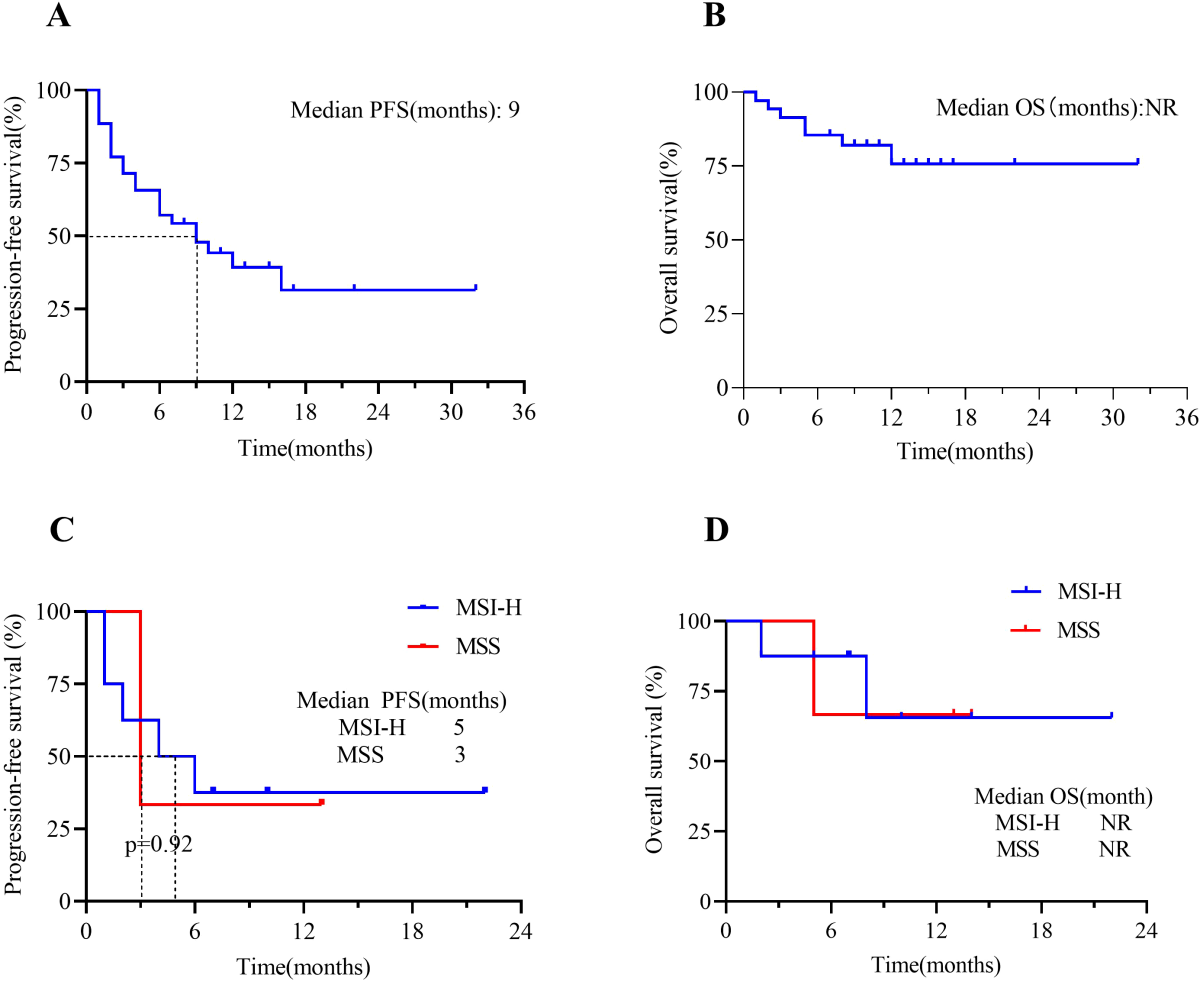
Kaplan-Meier analysis of patients treated with ICIs(n=35). **(A)** PFS in the overall population and **(B)** OS in the overall population; **(C)** PFS in EC patients who were in MSI-H vs. MSS groups; and **(D)** OS in EC patients who were in MSI-H vs. MSS groups. Tick marks represent censored patients. NR, not reached; PFS, progression-free survival; OS, overall survival; MSI-H, microsatellite instability-high; MSS, microsatellite stable.

**Figure 4:**
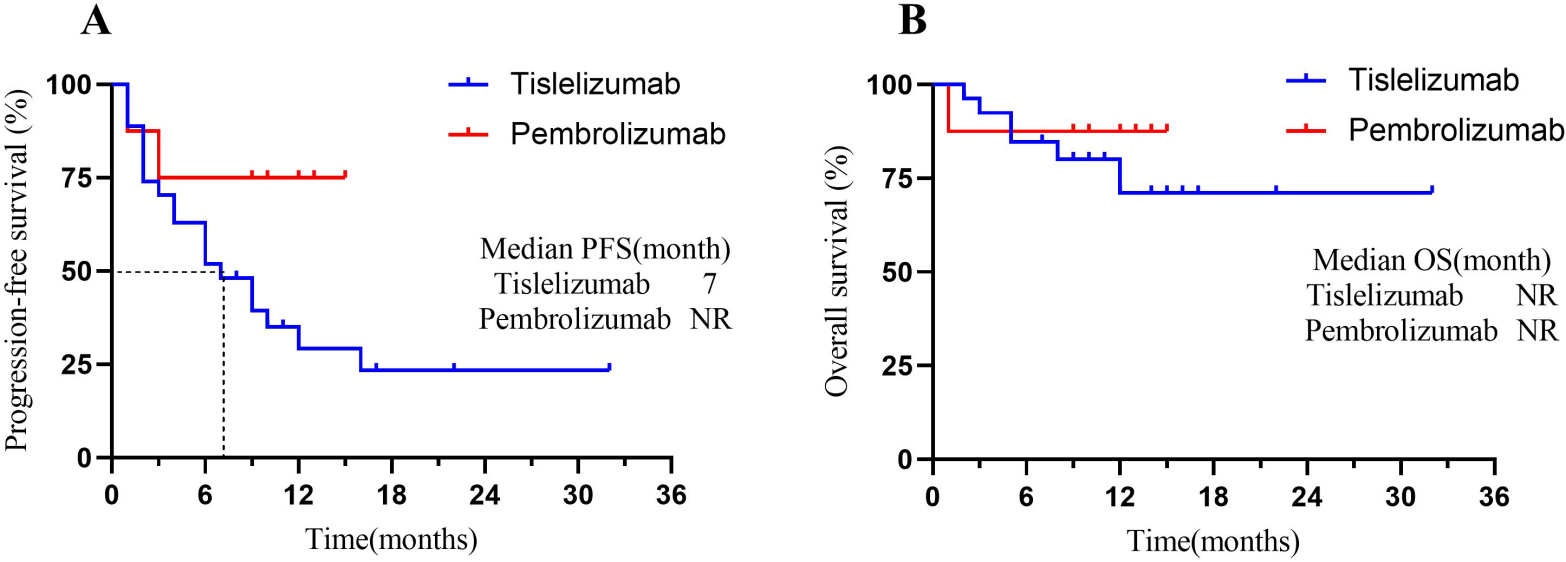
Kaplan-Meier analysis of patients treated with different types of ICIs. **(A)** PFS in patients who underwent Pembrolizumab vs. Tislelizumab groups; and **(B)** OS in patients who underwent Pembrolizumab vs. Tislelizumab groups. Tick marks represent censored patients. NR, not reached; PFS, progression-free survival; OS, overall survival.

At the time of data cutoff, 21 (60%) patients in the total population had experienced disease progression or death. The median PFS was 9 months (95% CI, 4 to 10), and the estimated PFS rates at 6 and 12 months were 57.1% and 39.3%, respectively (**Figure 3A**). Seven patients (20.0%) in the total population had died. In the total population, the median OS had not been reached [95% CI, 8 to not evaluable (NE)] (**Figure 3B**). The OS rate at 12 months was 75.7% (95% CI, 58.8% to 92.6%). In the endometrial cancer group, the median PFS in MSI-H patients was 5 months versus 3 months in MSS patients (Δ = 2 months, p=0.92) (**Figure 3C**); the median OS in both MSI-H and MSS patients had not been NR (p=0.89) (**Figure 3D**). When we focus on using different types of ICIs, the median PFS in the Tislelizumab group was 7 months. In contrast, the median PFS in the Pembrolizumab group had not been reached (p=0.09) (**Figure 4A**). The median OS in the Tislelizumab and Pembrolizumab groups had not been reached (p=0.54) (**Figure 4B**).

## Discussion

Using real-world data in an observational retrospective study, we summarized the frequency of MSI-H among patients with gynecological cancers in our single gynecologic center. Further, we analyzed patients with advanced or recurrent gynecological tumors receiving immunosuppressive therapy. We observed that the overall ORR was 34.3%, and 41.7% (5/12) of patients with an objective response had a complete response. Most importantly, tumor responses were durable; the median duration of response had not yet been reached. The OS outcomes were encouraging; the median OS time of patients with advanced or recurrent gynecological tumors treated with ICIs had not been reached, with an estimated 12-month OS rate of 75.7%.

After the FDA approval of pembrolizumab, an anti-PD-1 immune checkpoint monoclonal antibody, for the treatment of MSI-H/dMMR patients with unresectable or metastatic solid tumors in 2017, regardless of age and histological type (20). The exploration of MSI status as a predictive biomarker has been carried out in various tumor types. Thus, evaluating the MSI-H status in different types of solid tumors is vital. Large-scale analyses have shown a low incidence (3.5%-3.8%) of MSI-H in all the cancer types (13, 21). Our findings are in accord with recent studies that the frequency of MSI-H was 21.4%, and endometrial/uterine cancer has the highest MSI-H frequency, 25.7% among gynecologic cancers, while the frequency of MSI-H was 1.3% in cervical/vulvar/vagina cancer (14). In contrast, the frequency of ovarian/tubal/peritoneal cancers was 10.5% (2/19), higher than those reported in previous studies (14, 28). A possible explanation might be this study’s small number of cases and the lack of focused pathological review. However, a previous study reported MSI-H rates of 7.9% and 13.2% for ovarian cancer, which used the two PCR-based MSI methods. This suggests that differences in MSI analysis methods may affect these results (29).

When classified by histological type, the highest frequency of MSI-H was in dedifferentiated carcinomas, with 52.9%. There are similarities between the current study and ours in that half or more of dedifferentiated carcinomas lack mismatch repair proteins, and the percentage with deficiency ranges from 53% to 75% (30, 31). Dedifferentiated endometrial carcinoma is an uncommon and aggressive malignancy with a poor prognosis that is frequently misdiagnosed. Although its cause is usually unknown, it is thought to be related to Lynch syndrome (32). There is no data on the response rate of dedifferentiated cancers to ICIs, so the treatment of MSI-H/dMMR dedifferentiated cancers with ICIs may be considered. Contrary to earlier findings (14), which reported the frequency of MSI-H/dMMR in uterine carcinosarcoma was as low as 3.5%, the frequency of MSI-H was 23.1% (6/26) in carcinosarcoma in our study, which was relatively higher.

In other studies, the percentage of endometrial cancer molecular classification was found to be POLEmut (7.7%-12%), MMRd (28.1%-34.8%), p53abn (12.2%-23%), NSMP (32%-50.4%), respectively; compared to them, the results of endometrial cancer molecular typing in our center had a low percentage of POLEmut (6.9%), MMRd (26.6%), and P53abn (10.5%) were underrepresented, while NSMP (56%) was overrepresented (33–35). As the six endometrial cancers that received immunotherapy had molecular classification results, and five were MMRd type, they met the recommendations of the National Comprehensive Cancer Network (NCCN) guidelines. They were sensitive to immunotherapy (pembrolizumab can be used in patients with advanced/MSI-H cancers) (36). We found a case of endometrial cancer with molecular typing of p53 aberrant subtype that was maintained in SD status after treatment with Pembrolizumab, still alive at the end of follow-up, with a follow-up time of 13 months. Typically, the p53abn subtype responds poorly to immunotherapy. One study showed an ORR of 13% for pembrolizumab in PD-L1-positive (including p53 mutant) endometrial cancer, but limited efficacy in the p53abn subgroup (16). However, immunotherapy (e.g., pembrolizumab) may be considered if MSI-H/dMMR or high TMB is present.

In the MSI-H/dMMR environment, the appearance of neoantigens is further increased, thereby activating immune activity in the body (37). Furthermore, CD8+ T cell activity was promoted, and apoptosis of tumor cells was further increased when PD-1 inhibitors were used in the MSI-H/dMMR tumor environment (38). The therapeutic value of PD-1/PD-L1 inhibitor monotherapy in MSI-H/dMMR has been confirmed in clinical trials and is currently being used in clinical practice. A meta-analysis of 14 studies, including 939 MSI-H cancer patients, reported a pooled ORR for ICI of 41.5% (95% CI, 34.9% to 48.4%) and a pooled DCR of 62.8% (95% CI, 34.9% to 48.4%). The pooled median PFS was 4.3 months (95% CI, 3.0 to 6.8), and the pooled median OS was 24 months (95% CI, 20.1 to 28.5) (39). However, the effectiveness of immunotherapy varies due to economic and medical conditions in different countries and regions. The Keynote-158 clinical trial showed the ORR for MSI-H patients in all tumor types was 34.3% (95% CI, 28.3% to 40.8%), 57.1% for endometrial, and 33.3% for ovarian cancers (6). In a Korean multi-center study, the ORR was 21.6% (8/37) for cervical cancer, 4.7% (2/43) for ovarian cancer, and 25.8% (8/31) for endometrial cancer (15). A recent study conducted in China has discovered that when evaluating Tislelizumab monotherapy in patients with MSI-H/dMMR solid tumors, the ORR was 53.3%, with an ORR of 46.2% (6/13) in patients with endometrial cancer and a DCR of 53.8% (7/13). One case of cervical cancer and one case of ovarian cancer both achieved PRs (19).

Although it is difficult to compare the results of this study with those of clinical trial studies, we observed that the ORR of MSI-H gynecological tumors did not differ significantly from that of the overall population, with an ORR for the total number of patients (n=35) of 34.3%. In contrast, the ORR for the MSI-H group (n=11) was 36.4% (**Table 2**). Then, we further focused on endometrial cancer, which has a high frequency of MSI-H. However, contrary to the finding of Marabelle, who reported a much higher ORR (57.1%) and longer median PFS (25.7 months) in MSI-H endometrial cancer (6), the ORR of MSI-H endometrial cancer in our study is 25.2%. Surprisingly, the high ORR (66.7%) was observed in cervical cancers with MSI-H status. We speculate this might be due to the low incidence of MSI-H in cervical cancers. Two of the only three cervical cancer patients with MSI-H who were treated with ICI reached ORR. Most importantly, our study found that the tumor response was durable: the median response duration had not been reached, consistent with previous studies (6, 17, 39). Compared with some findings, our median PFS was longer, the median PFS of the total population is 9 months (95% CI, 4 to 10), probably because not all of our center’s treatment regimens were immunosuppressive monotherapy regimens, and many patients received combination chemotherapy or targeted therapies, which resulted in slower disease progression (17, 19, 40). A recent study has also found that the combination therapy of pembrolizumab and lenvatinib provides a favorable outcome for 37.2% (35/94) of patients with recurrent endometrial cancer. This provides a new combination therapy strategy for MSS tumors (41). Although two of the MSS endometrial cancer group already had a PD response, the median PFS and median OS were not statistically significant in the MSI-H endometrial cancer group compared to the MSS endometrial cancer group. An explanation for this might be that endometrial cancer patients with MSS in our study were treated with ICIs due to their PD-L1-positive status. In the NRG-GY018 trial, both the dMMR and pMMR subgroups showed a significant improvement in PFS (HR 0.30 and 0.54, respectively, p<0.001) (42). Yan’s meta-analysis provides solid evidence to support the use of lenvatinib in combination with pembrolizumab in the treatment of endometrial cancer, especially in patients with pMMR/MSS status (43). Therefore, for patients with pMMR/MSS endometrial cancer, immunosuppressive agents in combination with targeted therapies (e.g., CDK4/6 inhibitors, anti-angiogenic agents) need to be explored to improve efficacy further.

Many studies have shown that both Tislelizumab and Pembrolizumab have similar efficacy in the immunotherapy of gynecological cancers (17, 19, 41, 44, 45). As in this study, the ORR for patients treated with Pembrolizumab and Tislelizumab was identical (37.5% vs. 33.3%), and the median PFS and median OS were not statistically significant between the two groups. Pembrolizumab is generally used in patients with PD-L1 positivity (CPS>1) or MSI-H/dMMR in Cervical Cancer (17). In contrast, Tislelizumab may be suitable for patients with high PD-L1 expression (CPS≥10) or those more sensitive to immunotoxicity in cervical cancer (19). However, due to the lack of head-to-head trials, there are some limitations in directly comparing the efficacy of the two based on the available clinical trial data.

To our knowledge, this study is a retrospective study of a relatively large cohort in a western China single gynecologic center to evaluate the frequency of MSI-H in various gynecological tumor types and the therapeutic use of ICIs in gynecological cancers. The incidence of MSI-H in our center was comparable to that reported in most studies, and the ORR of gynecological tumors for immunotherapy was moderately high, with a long duration of DOR and a prolonged median PFS, revealing a significant clinical benefit of ICIs for gynecological tumors. This study has important implications for immunotherapy to advanced/recurrent gynecological cancers, especially in EC; ICIs can be attempted as a second-line treatment even if the MSI test is MSS. Our study has some limitations; as a real-world single-center retrospective study, there are missing and unknown data issues, and potential confounders and biases were introduced. For example, there were no ovarian cancer patients and only two vulvar cancer patients who received immunotherapy. Most of the patients who received immunotherapy were due to PD-L1 positivity, and the sample size of MSS endometrial cancer patients who received immunotherapy was small. However, real-world studies on immunotherapy in gynecological tumors are scarce, especially comparing MSI status in endometrial cancer. Therefore, our future studies will expand the sample size through multicenter collaborations to improve the findings’ statistical significance and clinical applicability and explore the discovery of other biomarkers predictive of ICIs in treating gynecological cancers. In addition, the predictive value of molecular classification types such as TP53 and POLE mutations for endometrial cancer treatment response remains to be clarified. There is also a need for further optimization of combination therapy strategies (e.g., immunotherapy + anti-vascular therapy + PARP inhibitors) for patients with MSS.

## Conclusion

This study has shown high MSI-H frequencies for the three major types of gynecological cancers, the highest of which is endometrial. Our study demonstrates the clinical benefit of treatment with ICIs in patients with advanced or recurrent gynecologic cancer. In patients with endometrial cancer, the effects of immunotherapy are likely to be consistent between the MSI-H and MSS status.

## Data Availability

The original contributions presented in the study are included in the article/**Supplementary Material**. Further inquiries can be directed to the corresponding author/s.
