# Supplementary material for: Frequency of microsatellite instability in gynecologic cancers and the efficacy of immune checkpoint inhibitors treated: real-world data from a single gynecologic center
Source: Front Immunol. 2025 May 9;16:1567824. doi: 10.3389/fimmu.2025.1567824 (PMC12098365; doi:10.3389/fimmu.2025.1567824)
Supplement: Supplementary file 1 [file Table1.docx]

**Table S1** Descriptive characteristics of patients with gynecologic cancers (n=1333)

|  | MSI-H | MSS/MSI-L | Overall |
| --- | --- | --- | --- |
|  | (n=285) | (n=1048) | (n=1333) |
| **Age** |  |  |  |
| <50 | 70 (24.6%) | 314 (30.0%) | 384 (28.8%) |
| ≥50 | 215 (75.4%) | 734 (70.0%) | 949 (71.2%) |
| **Family history** |  |  |  |
| No/Unknown | 244 (85.6%) | 931 (88.8%) | 1175 (88.1%) |
| Yes | 41 (14.4%) | 117 (11.2%) | 158 (11.9%) |
| **Personal history** |  |  |  |
| No/Unknown | 279 (97.9%) | 1034 (98.7%) | 1313 (98.5%) |
| Yes | 6 (2.1%) | 14 (1.3%) | 20 (1.5%) |
| **Origin of cancer** |  |  |  |
| Cervical/Vulvar/Vagina cancer | 3 (1.1%) | 220 (21.0%) | 223 (16.7%) |
| Endometrial/Uterine cancer | 280 (98.2%) | 811 (77.4%) | 1091 (81.8%) |
| Varian/Peritoneal/Tubal cancer | 2 (0.7%) | 17 (1.6%) | 19 (1.4%) |
| **Histology** |  |  |  |
| Carcinosarcoma | 6 (2.1%) | 20 (1.9%) | 26 (2.0%) |
| Clear cell carcinoma | 5 (1.8%) | 22 (2.1%) | 27 (2.0%) |
| Dedifferentiated carcinoma | 9 (3.2%) | 8 (0.8%) | 17 (1.3%) |
| Endometrioid adenocarcinoma | 240 (84.2%) | 680 (64.9%) | 920 (69.0%) |
| Mixed adenocarcinoma | 20 (7.0%) | 37 (3.5%) | 57 (4.3%) |
| Mucinous carcinoma | 1 (0.4%) | 0 (0%) | 1 (0.1%) |
| Serous carcinoma | 1 (0.4%) | 53 (5.1%) | 54 (4.1%) |
| Squamous cell carcinoma | 3 (1.1%) | 152 (14.5%) | 155 (11.6%) |
| Aden squamous carcinoma | 0 (0%) | 18 (1.7%) | 18 (1.4%) |
| Endocervical adenocarcinoma | 0 (0%) | 36 (3.4%) | 36 (2.7%) |
| Endometrial stromal sarcoma | 0 (0%) | 1 (0.1%) | 1 (0.1%) |
| Immature teratoma | 0 (0%) | 1 (0.1%) | 1 (0.1%) |
| Leiomyosarcoma | 0 (0%) | 1 (0.1%) | 1 (0.1%) |
| malignant melanoma | 0 (0%) | 1 (0.1%) | 1 (0.1%) |
| Mesonephric adenocarcinoma | 0 (0%) | 4 (0.4%) | 4 (0.3%) |
| Neuroendocrine carcinoma | 0 (0%) | 11 (1.0%) | 11 (0.8%) |
| undifferentiated carcinoma | 0 (0%) | 3 (0.3%) | 3 (0.2%) |
| **Grade** |  |  |  |
| G1/2 | 243 (85.3%) | 755 (72.0%) | 998 (74.9%) |
| G3/4 | 26 (9.1%) | 155 (14.8%) | 181 (13.6%) |
| No/Unknown | 16 (5.6%) | 138 (13.2%) | 154 (11.6%) |
| **FIGO stage** |  |  |  |
| I-II | 232 (81.4%) | 737 (70.3%) | 969 (72.7%) |
| III-IV | 53 (18.6%) | 311 (29.7%) | 364 (27.3%) |
| **LVSI** |  |  |  |
| No/Unknown | 173 (60.7%) | 740 (70.6%) | 913 (68.5%) |
| Yes | 112 (39.3%) | 308 (29.4%) | 420 (31.5%) |
| **Depth of invasion** |  |  |  |
| Deep | 69 (24.2%) | 293 (28.0%) | 362 (27.2%) |
| No/Unknown | 5 (1.8%) | 150 (14.3%) | 155 (11.6%) |
| Superficial | 211 (74.0%) | 605 (57.7%) | 816 (61.2%) |
| **PD-L1** |  |  |  |
| ＜1 | 3 (1.1%) | 20 (1.9%) | 23 (1.7%) |
| ≥1 | 6 (2.1%) | 218 (20.8%) | 224 (16.8%) |
| No/Unknown | 276 (96.8%) | 810 (77.3%) | 1086 (81.5%) |

Data are presented as No. (%) unless otherwise noted. Abbreviations: MSI-H, microsatellite high; MSS, microsatellite stable; FIGO, International Federation of Gynecology and Obstetrics; **LVSI**, lymph vascular space invasion; PD-L1, programmed death-ligand 1.

Table S2 Molecular classification types in Endometrial cancer (n=1020)

| Molecular classification | n (%) |
| --- | --- |
| POLEmut | 71 (6.9) |
| MMRd | 271 (26.6) |
| NSMP | 571 (56.0) |
| p53abn | 107 (10.5) |

Abbreviations: POLEmut, POLE mutation; MMRd, MMR deficiency; NSMP, non-specific molecular profile; p53abn, p53 abnormality.
